# Impact of a T cell-based blood test for tuberculosis infection on clinical decision-making in routine practice

**DOI:** 10.1016/j.jinf.2006.11.002

**Published:** 2007-03

**Authors:** Sarah Gooding, Oni Chowdhury, Tim Hinks, Luca Richeldi, Monica Losi, Katie Ewer, Kerry Millington, Ruba Gunatheesan, Stefania Cerri, Jeremy McNally, Ajit Lalvani

**Affiliations:** a Tuberculosis Immunology Group, Nuffield Department of Medicine, University of Oxford, John Radcliffe Hospital, Oxford OX3 9DU, UK; b Section of Respiratory Disease, Department of Oncology, Haematology, and Respiratory Disease, University of Modena and Reggio Emilia, Via del Pozzo, 71-41100 Modena, Italy; c Tuberculosis Immunology Group, Department of Respiratory Medicine, National Heart and Lung Institute, Wright Fleming Institute of Infection & Immunity, Imperial College London, Norfolk Place, London, W2 1PG, UK; d Department of General Medicine, Battle Hospital, Reading, RG30 1AG, UK

**Keywords:** Tuberculosis, Diagnosis, Blood test, Skin test

## Abstract

New T cell-based blood tests for tuberculosis infection could improve diagnosis of tuberculosis but their clinical utility remains unknown. We describe the role of the ELISpot test in the diagnostic work-up of 13 patients presenting with suspected tuberculosis in routine practice. Of the seven patients with a final diagnosis of active tuberculosis, all were positive by ELISpot including three with false-negative tuberculin skin test results. Rapid determination of tuberculosis infection by ELISpot accelerated the diagnosis of tuberculosis, enabling early treatment initiation.

## Introduction

Successful tuberculosis (TB) control depends on rapid detection and treatment of active cases, yet diagnosis is often difficult and delayed. Although sputum smear-positive pulmonary TB is readily diagnosed within hours, diagnosis of extra-pulmonary and smear-negative pulmonary TB can take much longer and bacteriological confirmation is often not possible.^1,2^

**Figure 1:**
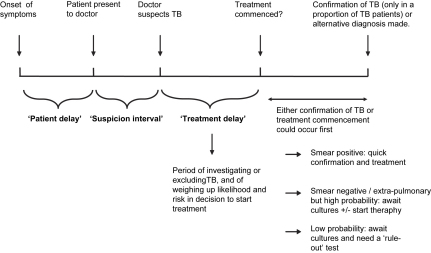
Demonstration of how periods of delay arise before treatment of active TB is initiated.

Causes for delay in initiation of TB treatment are multiple, and can be visualised along a timeline (Fig. 1). Studies examining health care system delays have shown that the longest delay is in deciding to initiate treatment.^3,4^ Treatment delay is common and prolonged because physicians have to make a presumptive diagnosis whilst still awaiting definitive culture results, knowing that these will frequently be falsely negative.

The tuberculin skin test (TST), which provides evidence of *M. tuberculosis* infection, is widely used to support clinical and radiological findings in the evaluation of patients with suspected tuberculosis.^5^ A positive TST result can help in the decision to start treatment while bacteriological confirmation is awaited or lacking. However, the greatest utility of a test of *M. tuberculosis* infection lies in its potential to exclude a diagnosis of TB when other tests are negative. This requires a test with high diagnostic sensitivity to minimise the possibility of falsely ruling out tuberculosis.

The poor sensitivity of TST in young children and immunosuppressed people makes it impossible to interpret negative TST results in these groups. Moreover, up to 25% of immunocompetent adults with active tuberculosis may have a negative or ambiguous result.^6^

A recently developed rapid blood test for *M. tuberculosis* infection, the enzyme-linked immunospot assay (ELISpot), may offer a more accurate alternative to the TST. The assay detects T cells specific for antigens in *M. tuberculosis*, which are absent from BCG and most environmental bacteria.^7^ It is therefore not confounded by prior BCG vaccination and is more specific than TST.^7–9^ ELISpot was found to have a sensitivity of 96% in HIV-negative adults with culture-confirmed active TB,^7^ and 92% in HIV-positive patients with pulmonary TB.^10^ These attributes suggest that ELISpot could improve and accelerate the diagnostic evaluation of patients with suspected TB.

The regulatory-approved, commercially available ELISpot assay, T-SPOT.*TB* (Oxford Immunotec Ltd, Abingdon, UK) is based on the assay used here, developed by Lalvani.^7–10^ T-SPOT.*TB* and an alternative interferon-gamma release assay, the whole blood ELISA (QuantiFERON-Gold, Cellestis, Carnegie, Australia), have recently been approved by the National Institute for Clinical Excellence (NICE) for diagnosis of latent TB infection and as adjunctive tests for the evaluation of patients with active TB. However, clinical data on the performance of these tests in TB patients and how they will impact on clinical decision-making is limited. The most relevant target population is that in which the test will actually be used in practice: patients presenting with suspected TB whose diagnoses are unknown at the time of testing.^11,12^ We therefore monitored the diagnostic performance of ELISpot in patients with suspected TB in routine clinical practice.

We received requests to perform ELISpot assays from physicians at two institutions who were aware of the published literature on ELISpot. The decision to request an ELISpot blood test was made by the referring physicians and arose from their routine clinical practice. To date, 13 patients with suspected active TB have been tested and we report the comparative performance of ELISpot and TST and discuss how the availability of ELISpot results enabled early initiation of treatment.

## Methods

### Study population

Six child and seven adult inpatients from the Departments of Pulmonary Medicine and Paediatrics, Modena University Hospital, Italy, and the Department of General Internal Medicine, Battle Hospital, Reading, UK, being investigated for suspected tuberculosis, were referred for ELISpot testing. None had symptoms or signs of HIV infection or were suspected of HIV carriage, and all were from populations at low risk of HIV. Of the 13, seven had a final diagnosis of active tuberculosis (Tables 1 and 2). All patients, or their parents, gave verbal informed consent to have an ELISpot blood test. Ethical approval was granted by Central Oxford Research Ethics Committee, Modena University Hospital Ethics Committee and the Battle Hospital Ethics Committee.

### Procedures

ELISpot was performed as previously described using 35 overlapping peptides spanning the length of ESAT-6 and CFP10 arranged in six pools and 250,000 peripheral blood mononuclear cells were added to each ELISpot well.^9^ All assays produced evaluable results. The assays were carried out within 1 week of initial presentation to the hospital. Both ELISpot and TSTs were performed during the diagnostic evaluation period before therapy was commenced. Twelve of the thirteen TSTs were performed using the Mantoux method in accordance with guidelines. Five international units of purified protein derivative (PPD) were injected intradermally and the tests were interpreted within 72 h. A positive result was defined as greater than 5 mm regardless of BCG status. Case 4 had a Heaf test performed via the standard method using a six-needle disposable Heaf gun injecting 100,000 units per ml of PPD, and was read 1 week afterwards.^9^ All tests were interpreted by an experienced physician.

## Results and discussion

Of the 13 patients, seven had a final diagnosis of active TB. Of these, two were culture-confirmed, one was confirmed by nucleic acid amplification and the remaining four were diagnosed on clinical and radiological grounds. All had an excellent response to anti-TB therapy (Table 1). Active TB was clinically and microbiologically excluded in the remaining six patients (Table 2). Of these, two were negative by TST and ELISpot (one with pneumonia, the other with bronchiectasis, cases 8 and 9); three were positive by TST and ELISpot and were treated for latent TB infection and one, a 7-month-old infant with recent TB exposure and respiratory symptoms, had a positive ELISpot but negative TST, and was treated for latent TB infection (case 13). All six have remained well over 18 months follow-up.

All seven active TB cases were ELISpot positive but three were TST negative at presentation. Of these three, case 1 presented with pyrexia and night sweats and had inconclusive radiological findings. Although TB was in the differential diagnosis, there was no definitive evidence, and acid fast stains and the skin test were negative. The positive ELISpot result, performed 3 days after the negative skin test, provided the only specific evidence for TB infection, making a diagnosis of TB much more likely and prompting early initiation of therapy which resulted in an excellent clinical response. Initiation of anti-TB treatment would otherwise likely have been delayed several weeks. Case 2 presented with constrictive pericarditis and pulmonary nodules. Although the clinical picture was compatible with TB, acid fast stains, culture of bronchoalveolar lavage fluid and pericardial biopsy tissue were negative, as was the skin test. A positive ELISpot result provided definitive evidence of TB infection, prompting the presumptive diagnosis of tuberculous pericarditis and initiation of anti-tuberculous chemotherapy just 4 days after admission. Case 7 was a heavy smoker presenting with cough and haemoptysis and two nodules in the right upper lung, a picture highly suggestive of lung cancer. However the positive ELISpot result—despite the negative TST result—suggested a diagnosis of pulmonary TB, then confirmed by surgical biopsy which revealed caseating granulomas and was positive on nucleic acid amplification.

Anergy is defined as the absence of the cutaneous delayed type hypersensitivity (DTH) response to PPD during active tuberculosis. These cases suggest that the ELISpot is less susceptible to this anergy, giving fewer false-negative results. None of the three cases with active TB and a negative skin test had intrinsic or iatrogenic immunosuppression to account for the negative skin test results. Therefore, these false-negative results were likely caused by the active tuberculosis per se. The ability of ELISpot to detect TB infection in the setting of anergic skin test results is likely due to the fact that a greater number of *M. tuberculosis* antigen specific Th1-type T cells are required to produce a cutaneous DTH response than an ELISpot response.^13^

Our report has several limitations. Only two of seven TB patients had a gold-standard culture-confirmed microbiological diagnosis. However, the clinical and radiological picture in the remaining cases was either compatible with or strongly suggestive of active TB and was further corroborated by the excellent response to anti-TB therapy in each case. Performance of ELISpot in patients with a clinical diagnosis of TB is highly relevant for clinical practice, where culture-confirmation is not possible in a significant proportion of patients.

Of the 13 patients with suspected TB, 11 had positive ELISpot results of whom seven were treated for active disease (Table 1). In the remaining four ELISpot-positive patients, active TB was clinically and microbiologically excluded and they were treated for latent TB infection; three of these patients also had positive TST results (Table 2). Thus, as expected for a test of infection, ELISpot did not help in differentiating active TB disease from latent TB infection, which remains a distinction based on clinical judgement.

The numbers are small but likely to be representative of patients presenting to hospital with suspected active TB in general, as they were referred from routine clinical practice. However, it is difficult to rule out possible selection bias employed by the requesting physicians. As collaborating physicians who were aware of the ELISpot test, they are also potentially not representative of the majority of doctors.

In summary, we have presented three cases of active tuberculosis with a negative tuberculin skin test and a positive ELISpot. In each of these cases the ELISpot assay enabled a diagnosis to be reached more rapidly than would have otherwise been possible, allowing rapid treatment initiation. The ELISpot has shown greater sensitivity as a diagnostic test compared to the TST in latent TB^9^ and active TB.^7^ Our preliminary findings suggest that this increased sensitivity can result in improved diagnostic evaluation of TB suspects with false-negative TST results in routine clinical practice.

## Figures and Tables

**Table 1:** Clinical characteristics and test results of the seven active tuberculosis cases

	Case 1	Case 2	Case 3	Case 4	Case 5	Case 6	Case 7
Age (years)	65	54	71	27	2	1.5	68

Sex	M	M	F	F	M	M	M

Clinical features	Swinging pyrexia and night sweats	Dyspnoea, constrictive pericarditis	Arthralgia, pyrexia and back pain	Weight loss, pyrexia and cough	Weight loss, pyrexia and cough	Productive cough and weight loss	Cough and haemoptysis

Radiology	Bilateral pleural effusions and ascites	Pericardial thickening and pulmonary miliary nodules on HRCT^a^	Para-aortic lymphadenopathy and enlarged right hilum on HRCT^a^	Bilateral nodular opacities in upper lobes. Nodular lesions and tree in bud on HRCT^a^	Hilar lymphadenopathy	Right hilar node enlargement and parenchymal upper lobe infiltrate	Two small nodules in right upper lung

Microbiology and histology	Sputum, pleural and ascitic fluid: ZN^b^ stain and culture negative. Pleural and ascitic fluid: lymphocytic exudates	Pericardial biopsy: granuloma. BAL^c^: ZN stain and culture negative	No samples obtained; biopsy refused	Sputum and BAL^c^: ZN^b^ stain and culture negative	Gastric washings: ZN^b^ stain negative, culture positive	Gastric washings: culture positive	Lung biopsy: granulomas with central necrosis

Basis for final diagnosis of TB	Clinical picture, response to therapy	Clinical and radiological picture, response to therapy	Clinical and radiological picture, response to therapy	Clinical and radiological picture, response to therapy	Culture confirmed	Culture confirmed	Positive nuclei acid amplification on tissue sample from lung

Site of disease	Pleural effusion + ascites	Pulmonary (miliary) + pericardial	Para-aortic lymph nodes	Pulmonary	Pulmonary	Pulmonary	Pulmonary

BCG vaccination status	BCG vaccinated	Unvaccinated	BCG vaccinated	Unvaccinated	Unvaccinated	Unvaccinated	Unvaccinated

Mantoux result	0 mm	0 mm	Heaf grade 4	10 mm	25 mm	9 mm	0 mm

ELISpot Results^d^	Positive	Positive	Positive	Positive	Positive	Positive	Positive

a High resolution computerised tomography.

b Ziel Nielson.

c Bronchioalveolar lavage.

d Duplicate pairs of wells were scored positive if they contained a mean of at least five spot-forming cells more than the mean of the negative control wells, and, in addition, this number was at least twice the mean of the negative control wells.

**Table 2:** Clinical characteristics and test results of the six cases without active tuberculosis

	Case 8	Case 9	Case 10	Case 11	Case 12	Case 13
Age	1 year	3 years	52 years	5 years	62 years	7 months

Sex	F	M	F	F	M	M

Clinical features	Respiratory symptoms; TB contact	Previously treated TB. Immigrant from TB endemic area	Cough and low grade fever	Cough and low grade fever. Previously treated for LTBI. Immigrant from TB endemic area	TB contact.	Respiratory symptoms. TB contact

Radiology	Pulmonary parenchymal consolidation	Fibrotic changes on chest x-ray	Small calcified nodule in upper left lobe	Normal chest x-ray	Fibrotic changes on chest x-ray	Pulmonary parenchymal consolidation

Microbiology and histology	Gastric and respiratory aspirate negative for *M. tuberculosis* (microscopy, PCR and culture); culture positive for *H. influenzae*	Gastric aspirate negative for *M. tuberculosis* (microscopy, PCR and culture)	Sputum and BAL negative for *M. tuberculosis*	Gastric aspirate negative for *M. tuberculosis* (microscopy, PCR and culture)	Sputum and BAL negative for *M. tuberculosis* (microscopy, PCR and culture)	Gastric aspirate negative for *M. tuberculosis* (microscopy, PCR and culture)

Basis for final exclusion of TB	Clinical response to antibiotic therapy for community-acquired pneumonia. Final diagnosis: bacterial pneumonia	Negative microbiology, no symptoms. Final diagnosis: previously treated TB leading to bronchiectasis	Clinical response to first-line antibiotics for community-acquired chest infection. Final diagnosis: LTBI^b^	Symptoms self-limiting. Final diagnosis: LTBI^b^	Final diagnosis: LTBI^b^	Clinical response to first-line antibiotics for community-acquired chest infection. Final diagnosis: LTBI^b^

Mantoux result	Negative (0 mm)	Negative (3 mm)	Positive (20 mm)	Positive (45 mm)	Positive (14 mm)	Negative (0 mm)

ELISpot results^a^	Negative	Negative	Positive	Positive	Positive	Positive

a Duplicate pairs of wells were scored positive if they contained a mean of at least five spot-forming cells more than the mean of the negative control wells, and, in addition, this number was at least twice the mean of the negative control wells.

b LTBI, latent TB infection. All patients diagnosed with LTBI remained well during 18 months follow-up without treatment for active TB.

## References

[1] Tuberculosis Section, Communicable Disease Surveillance Centre London. Preliminary report on tuberculosis cases reported in 2001 in England, Wales and Northern Ireland, March 2003. http://www.phls.org.uk/topics_az/tb/tbfrontpage.htm.

[2] Tuberculosis Section, Communicable Disease Surveillance Centre London. Annual report on tuberculosis cases reported in 2000 in England, Wales and Northern Ireland, March 2003. http://www.phls.org.uk/topics_az/tb/tbfrontpage.htm.

[3] Venkatarama K., Iademarco E.P., Fraser V.J., Kollef M.H. (1999). Delays in the suspicion and treatment of tuberculosis among hospitalised patients. Ann Intern Med.

[4] Sherman L.F., Fujiwara P.I., Cook S.V., Bazerman L.B., Frieden T.R. (1999). Patient and health care system delays in the diagnosis and treatment of tuberculosis. Int J Tuberc Lung Dis.

[5] Huebner R.E., Schein M.F., Bass J.B. (1993). The tuberculin skin test. Clin Infect Dis.

[6] Nash D.R., Douglass J.E. (1980). Anergy in active pulmonary tuberculosis. Chest.

[7] Lalvani A., Pathan A.A., McShane H., Wilkinson R.J., Latif M., Conlon C.P. (2001). Rapid detection of *Mycobacterium tuberculosis* infection by enumeration of antigen-specific T cells. Am J Respir Crit Care Med.

[8] Lalvani A., Pathan A.A., Durkan H., Wilkinson K.A., Deeks J., Reece W.H. (2001). Enhanced contact tracing and spatial tracking of *Mycobacterium tuberculosis* infection by enumeration of antigen-specific T cells. Lancet.

[9] Ewer K., Deeks J., Alvarez L., Bryant G., Waller S., Anderson P. (2003). Comparison of T-cell-based assay with tuberculin skin test for diagnosis of *Mycobacterium tuberculosis* infection in a school tuberculosis outbreak. Lancet.

[10] Chapman A.L., Munkanta M., Wilkinson K.A., Pathan A.A., Ewer K., Ayeles H. (2002). Rapid detection of active and latent tuberculosis infection in HIV-positive individuals by enumeration of *Mycobacterium tuberculosis*-specific T cells. AIDS.

[11] Arend S.M., Ottenhoff T.H.M., Andersen P., Van Diesel J.T. (2001). Uncommon presentations of tuberculosis: the potential value of a novel diagnostic assay based on the *Mycobacterium tuberculosis*-specific antigens ESAT-6 and CFP-10. Int J Tuberc Lung Dis.

[12] Small M., Perkins P. (2000). More rigour needed in trials of new diagnostic agents for tuberculosis. Lancet.

[13] Richeldi L., Ewer K., Losi M., Bergamini B.M., Roversi P., Deeks J. (2004). T cell-based tracking of multidrug resistant tuberculosis infection after brief exposure. Am J Respir Crit Care Med.

